# The predictive value of D-dimer combined with systemic immune-inflammation index for the presence of pulmonary thromboembolism in AECOPD patients

**DOI:** 10.3389/fmed.2025.1582913

**Published:** 2025-08-01

**Authors:** Xuanna Zhao, Jiahua Li, Yunan Wang, Bangxiao Huang, Xiaobing Xie, Min Chen, Bin Wu, Dan Huang, Dongming Li, Dong Wu

**Affiliations:** Department of Respiratory and Critical Care Medicine, Affiliated Hospital of Guangdong Medical University, Zhanjiang, China

**Keywords:** acute exacerbations of chronic obstructive pulmonary disease, pulmonary thromboembolism, D-dimer, systemic immune inflammatory index, predictive model

## Abstract

**Objective:**

This study aims to evaluate the predictive potential of D-dimer levels and the systemic immune-inflammatory index (SII) for identifying concurrent pulmonary thromboembolism (PTE) in patients with acute exacerbations of chronic obstructive pulmonary disease (AECOPD).

**Methods:**

We conducted a case–control study involving 75 patients with AECOPD and concurrent PTE, admitted to the Affiliated Hospital of Guangdong Medical University between June 2017 and December 2020. A control group comprising 76 AECOPD patients without PTE was included for comparison. Clinical characteristics and laboratory findings were compared between the two groups. Multivariate logistic regression was employed to identify independent risk factors for PTE in AECOPD patients. The predictive accuracy of these risk factors was assessed using receiver operating characteristic (ROC) curves, while Spearman correlation analysis evaluated associations between variables. Model robustness was validated internally using bootstrap techniques, and decision curve analysis (DCA) was applied to determine the clinical utility of the predictive model.

**Results:**

Multivariate logistic regression identified D-dimer [odds ratio (OR) 1.17, 95% confidence interval (CI) 1.03–1.38] and the SII (OR 1.003, 95% CI 1.000–1.006) as independent risk factors for PTE in patients with AECOPD. The area under the ROC curve (AUC) values for predicting PTE in AECOPD patients were 0.758 (95% CI 0.682–0.834) for D-dimer, 0.757 (95% CI 0.677–0.838) for SII, and 0.834 (95% CI 0.768–0.900) for their combination, respectively. The Bootstrap results demonstrated that the model combining D-dimer and SII had an AUC of 0.8409 (95% CI: 0.7649, 0.9156), indicating robust performance across resampled datasets. Both D-dimer and SII showed a positive correlation with hospital stay duration (*r* = 0.289, *p* = 0.047; *r* = 0.235, *p* = 0.043). DCA demonstrated that the combination of D-dimer and SII provides significant net benefit in clinical decision-making for predicting PTE in AECOPD patients.

**Conclusion:**

D-dimer and SII are independent risk factors for predicting concurrent PTE in AECOPD patients. The combination of these two markers demonstrates robust predictive accuracy, and their levels may be positively correlated with disease severity. These findings suggest that D-dimer and SII could play a valuable role in clinical decision-making and risk stratification in AECOPD patients.

## Introduction

Chronic Obstructive Pulmonary Disease (COPD) is a common chronic condition marked by persistent airflow limitation, typically associated with chronic bronchitis and/or emphysema. As the disease progresses, patients may develop severe complications, such as pulmonary thromboembolism (PTE), pulmonary heart disease and respiratory failure (1, 2). The prevalence and mortality rates of COPD remain high globally, posing a significant economic burden and exerting substantial pressure on healthcare systems.

During acute exacerbations of chronic obstructive pulmonary disease (AECOPD), patients may experience a rapid deterioration of respiratory symptoms, requiring additional pharmacological treatment or hospitalization. At this stage, both pro-inflammatory and anti-inflammatory responses are triggered, and the blood’s tendency to clot is further heightened (3, 4). Consequently, AECOPD is recognized as an independent risk factor for the development of PTE (5). Conversely, PTE negatively impacts the prognosis of AECOPD, leading to prolonged hospital stays and increased mortality rates among affected patients (6). The interaction between AECOPD and PTE often accelerates the progression of the disease. However, due to the high symptom overlap between the two, timely identification of concomitant PTE becomes increasingly challenging, potentially leading to delayed treatment. Therefore, the identification of reliable biomarkers to accurately predict the risk of PTE in AECOPD patients is of paramount importance.

D-dimer is commonly utilized as an initial diagnostic tool for suspected acute PTE, with its negative value playing a key role in ruling out thrombotic conditions. The 2019 European guidelines recommend D-dimer testing for patients with a low to moderate probability of PTE to reduce unnecessary imaging evaluations (7). However, previous studies have shown that D-dimer levels are elevated in patients with AECOPD compared to those with stable COPD, complicating its diagnostic accuracy for detecting PTE in this population (8), so that it is not sufficient to rely solely on D-dimer to distinguish between AECOPD patients with PTE (9). Therefore, this study aims to explore potential novel biomarkers that may enhance the diagnostic accuracy of PTE in patients with AECOPD, with the goal of reducing reliance on imaging techniques.

SII is a comprehensive marker of systemic inflammation and immune status, derived from peripheral blood neutrophils, lymphocytes, and platelets (10). SII is calculated by multiplying the platelet count by the neutrophil-to-lymphocyte ratio (NLR) and has been widely utilized as a prognostic indicator in cancer research (10). Recent studies have shown a positive correlation between elevated SII and an increased risk of COPD, suggesting it may be a more effective predictor of COPD risk compared to other inflammatory markers such as NLR and the platelet-to-lymphocyte ratio (PLR) (11). Nonetheless, there is a paucity of research investigating the association between the SII and PTE in the context of AECOPD. D-dimer serves as a biomarker for thrombus formation, whereas SII is indicative of the systemic inflammatory state. The integration of these two biomarkers may enhance the precision in diagnosing pulmonary embolism and other inflammatory conditions (12, 13). Thus, this study investigates the effectiveness of combining D-dimer and SII for identifying PTE in AECOPD patients.

## Methods

### Data source

The data were obtained from the COPD-AD China Registry, a national clinical registry study focused on COPD with anxiety and depression, which began in June 2017 and lasted for 3.5 years (clinical trial ID: NCT03187236) (14). This multicenter, prospective, cohort study was approved by the central independent ethics committee (Fuwai Hospital, Approval Number: 2017–879, date of approval: October 21, 2016), and complied with the Declaration of Helsinki., and written informed consent was obtained from all enrolled patients.

### Study population

We retrieved data from the electronic medical record system of the Affiliated Hospital of Guangdong Medical University, identifying 75 patients diagnosed with AECOPD and concurrent PTE. These patients were matched with a control group of 76 patients diagnosed with AECOPD alone. Data were collected between June 2017 and December 2020. Inclusion criteria were as follows: (1) age over 40 years; (2) AECOPD, defined as an acute worsening of respiratory symptoms requiring additional treatment in patients diagnosed with COPD according to the 2017 Global Initiative for Chronic Obstructive Lung Disease (GOLD) (15); (3) completion of computed tomography pulmonary angiography (CTPA) during hospitalization; (4) no use of oral or intravenous antibiotics, antiplatelet agents, or anticoagulants within 3 months prior to admission. Exclusion criteria encompassed patients with bronchial asthma, bronchiectasis, interstitial lung disease, tuberculosis, acute myocardial infarction or stroke, severe liver or renal dysfunction, malignancy, hematological disorders, or rheumatic autoimmune diseases.

### Data collection

Demographic data, including gender, age, smoking history, and length of hospital stay, as well as clinical comorbidities such as PTE, lower extremity venous thrombosis, and arrhythmia, were collected. Laboratory parameters included peripheral blood counts (white blood cells, neutrophils, red blood cells, lymphocytes, monocytes, and platelets), D-dimer levels, N-terminal pro-brain natriuretic peptide (NT-proBNP), high-sensitivity troponin T, fibrinogen, serum creatinine, serum uric acid, urea, cystatin C, low-density lipoprotein (LDL), high-density lipoprotein (HDL), triglycerides, total cholesterol, SII, systemic inflammation response index (SIRI), neutrophil-to-lymphocyte ratio (NLR), platelet-to-lymphocyte ratio (PLR), and pulmonary artery computed tomography angiography (CTA).

### Statistical analysis

All statistical analyses were conducted using IBM SPSS version 26.0 and R Studio version 4.4.1. The distribution of continuous variables was evaluated with the Kolmogorov-Smirnov (K-S) normality test. Normally distributed continuous variables were reported as the mean ± standard deviation (SD) and compared using independent sample t-tests. Non-normally distributed continuous variables were presented as the median (interquartile range, IQR) and analyzed with Mann–Whitney U tests. Categorical variables were expressed as percentages (%) and compared using chi-square tests. Spearman correlation analysis was used to assess relationships between variables. Logistic regression analysis was performed to identify risk factors for AECOPD with PTE. Bootstrap Internal Validation was employed to assess the model’s robustness. Decision curve analysis (DCA) was applied to evaluate the net benefit of the predictive model across various risk thresholds. Statistically significant variables identified through logistic regression were incorporated into a receiver operating characteristic (ROC) curve to assess diagnostic performance. A *p*-value < 0.05 was considered statistically significant.

## Results

### Comparison of general characteristics between the AECOPD group and AECOPD complicated with PTE group

The results indicated that patients in the AECOPD group with PTE had a significantly higher incidence of arrhythmias and lower extremity venous thrombosis compared to those in the AECOPD-alone group (*p* < 0.005). However, no statistically significant differences were observed between the two groups regarding age, gender, length of hospital stay, or smoking history (*p* > 0.05). Detailed results are provided in Table 1.

**Table 1:** Comparison of general data between AECOPD group and AECOPD complicated with PTE group.

General characteristics	AECOPD complicated with PTE group (*n* = 75)	AECOPD group (*n* = 76)	*p* value
Male, *n* (%)	57 (76%)	63 (82.9%)	0.397
Female, *n* (%)	18 (24%)	13 (17.1%)	0.296
Smoking history, *n* (%)	38 (50.7%)	50 (65.8%)	0.086
Age (years), mean ± SD	75.61 ± 8.57	74.74 ± 10.55	0.576
Hospital stays (days), mean ± SD	16.60 ± 17.27	14.84 ± 15.35	0.485
Arrhythmias, *n* (%)	36 (52%)	27 (35.5%)	0.061
Lower extremity venous thrombosis, *n* (%)	29 (39.2%)	1 (1.3%)	<0.001*

Data presented as mean ± SD and percentage (%); *: *p* < 0.05; AECOPD, acute exacerbation of chronic obstructive pulmonary disease; PTE, pulmonary thromboembolism.

### Comparison of laboratory indicators between the AECOPD group and AECOPD complicated with PTE group

Given that inflammatory status, cardiovascular diseases, renal insufficiency, arrhythmia and lower extremity venous thrombosis are associated with risk factors for pulmonary embolism in acute exacerbation of chronic obstructive pulmonary disease, we selected these indicators as laboratory parameters (16–18). In the AECOPD group with PTE, white blood cell count, neutrophil count, D-dimer, urea, cystatin C, NT-ProBNP, high-sensitivity troponin T, SII, SIRI, NLR, and PLR were significantly higher compared to the AECOPD-alone group (*p* < 0.05). Conversely, lymphocyte count, LDL, and total cholesterol were significantly lower in the AECOPD with PTE group (*p* < 0.05). No statistically significant differences were found between the two groups in monocyte count, fibrinogen, serum creatinine, serum uric acid, triglycerides, red blood cell count, platelet count, or HDL (*p* > 0.05). Detailed results are presented in Table 2.

**Table 2:** Comparison of laboratory indicators between the AECOPD group and AECOPD complicated with PTE group.

Laboratory indicators	AECOPD complicated with PTE group (*n* = 75)	AECOPD group (*n* = 76)	*p* value
White blood cell count (K/uL), mean ± SD	11.23 ± 9.34	7.04 ± 2.29	<0.001*
Neutrophil count (K/uL), mean ± SD	8.53 ± 4.17	4.90 ± 1.85	<0.001*
Lymphocyte count (K/uL), mean ± SD	0.96 ± 0.64	1.32 ± 0.59	<0.001*
Red blood cell count (10^3^ K/uL), mean±SD	4.30 ± 0.91	4.32 ± 0.71	0.911
Monocyte count (K/uL), mean ± SD	0.64 ± 0.38	0.60 ± 0.28	0.513
Platelet count (K/uL), mean ± SD	214.10 ± 96.34	234.61 ± 82.96	0.163
Neutrophil-to-lymphocyte ratio (%), mean ± SD	14.73 ± 15.04	4.37 ± 2.27	<0.001*
Platelet-to-lymphocyte ratio (%), mean ± SD	323.40 ± 255.09	208.32 ± 116.59	<0.001*
D-dimer (μg/mL), mean ± SD	6.86 ± 7.82	2.24 ± 2.60	<0.001*
SII, mean ± SD	2889.42 ± 3098.31	1030.14 ± 654.25	<0.001*
SIRI, mean ± SD	8.58 ± 10.32	2.63 ± 1.91	<0.001*
Fibrinogen (g/L), mean ± SD	8.84 ± 20.30	3.94 ± 1.46	0.037*
Serum creatinine (umol/L), mean ± SD	78.13 ± 34.16	72.20 ± 23.33	0.214
Uric acid (umol/L), mean ± SD	321.64 ± 151.66	282.24 ± 95.37	0.058
Urea (mmol/L), mean ± SD	7.54 ± 5.25	5.40 ± 2.26	0.001*
Cystatin C (mg/L), mean ± SD	1.38 ± 2.33	0.94 ± 0.33	0.103
NT-proBNP (pg/mL), mean ± SD	3541.81 ± 5601.41	624.81 ± 1146.98	<0.001*
High-sensitivity troponin T (ng/mL), mean ± SD	0.04 ± 0.06	0.02 ± 0.02	0.002*
Low-density lipoprotein (mmol/L), mean ± SD	2.51 ± 1.03	2.87 ± 1.12	0.043*
High-density lipoprotein (mmol/L), mean ± SD	1.25 ± 0.48	1.34 ± 0.45	0.201
Triglycerides (mmol/L), mean ± SD	1.07 ± 0.50	1.03 ± 0.79	0.693
Total cholesterol (mmol/L), mean ± SD	4.24 ± 1.31	4.64 ± 1.26	0.058

Data presented as mean ± SD; *: *p* < 0.05; AECOPD, acute exacerbation of chronic obstructive pulmonary disease; PTE, pulmonary thromboembolism; SII, systemic immune-inflammatory index; SIRI, system inflammation response index; NT-proBNP, N-terminal pro-brain natriuretic peptide.

### Logistic regression analysis of AECOPD complicated with PTE

Univariate logistic regression analysis was initially conducted with AECOPD combined with PTE as the dependent variable, and the clinically significant variables with statistically significant differences based on Tables 1, 2 as independent variables. The results revealed that white blood cell count, neutrophil count, lymphocyte count, D-dimer, urea, NT-ProBNP, high-sensitivity troponin T, SII, SIRI, NLR, PLR, LDL, total cholesterol, arrhythmias, and lower extremity venous thrombosis were significantly associated with AECOPD with PTE. Multivariate binary logistic regression analysis was then conducted using clinically significant variables identified from the univariate analysis. The results indicated that D-dimer, SII, lymphocyte count and the presence of lower extremity venous thrombosis were independent risk factors for AECOPD with PTE. Specifically, D-dimer [odds ratio (OR) = 1.17, 95% confidence interval (CI): 1.03–1.38, *p* = 0.039] and SII (OR = 1.003, 95% CI: 1.000–1.006, *p* = 0.046) had OR greater than 1, signifying that as D-dimer and SII levels increase, the likelihood of concurrent PTE in AECOPD patients also rises. Further details are provided in Table 3.

**Table 3:** Analysis of influencing factors of AECOPD complicated with PTE.

Variable	Univariate logistic regression analysis OR (95% CI)	*p* value	Multivariate logistic regression analysis OR (95%CI)	*p* value
White blood cell count (K/uL)	1.37 (1.21–1.59)	<0.001*	1.06 (0.98–1.14)	0.476
Neutrophil count (K/uL)	1.55 (1.33–1.86)	<0.001*	0.64 (0.26–1.19)	0.178
Lymphocyte count (K/uL)	0.36 (0.19–0.65)	0.001*	7.23 (1.47–38.87)	0.012*
D-dimer (μg/mL)	1.27 (1.14–1.45)	<0.001*	1.17 (1.03–1.38)	0.039*
Urea (mmol/L)	1.21 (1.08–1.4)	0.003*	1.13 (0.88–1.53)	0.425
NT-ProBNP (pg/mL)	1.000 (1.000–1.001)	<0.001*	1.000 (1.000–1.0001)	0.165
High-sensitivity troponin T (ng/mL)	6.94*10^17 (1.91*10^9–8.95*10^27)	<0.001*	1.82*10^8 (0.040–8.90*10^18)	0.145=
SII	1.0009 (1.0006–1.001)	<0.001*	1.003 (1.000–1.006)	0.046*
SIRI	1.41 (1.23–1.68)	<0.001*	1.03 (0.74–1.58)	0.871
Neutrophil-to-lymphocyte ratio	1.34 (1.21–1.53)	<0.001*	1.55 (1.15–2.35)	0.267
Platelet-to-lymphocyte ratio	1.005 (1.001–1.012)	0.001*	0.99 (0.97–1.00)	0.251
Low-density lipoprotein (mmol/L)	0.73 (0.53–0.99)	0.046*	0.65 (0.13–3.45)	0.601
Total cholesterol (mmol/L)	0.78 (0.6–1.01)	0.06	0.87 (0.20–3.43)	0.844
Arrhythmias	1.97 (1.03–3.81)	0.042*	2.10 (0.65–7.19)	0.221
Lower extremity venous thrombosis	48.33 (9.81–875.82)	<0.001*	47.44 (6.41–1060.57)	0.001*

*: *p* < 0.05; AECOPD, acute exacerbation of chronic obstructive pulmonary disease; PTE, pulmonary thromboembolism; NT-proBNP, N-terminal pro-brain natriuretic peptide; SII, systemic immune-inflammatory index; SIRI, system inflammation response index.

### ROC curve analysis

**Figure 1:**
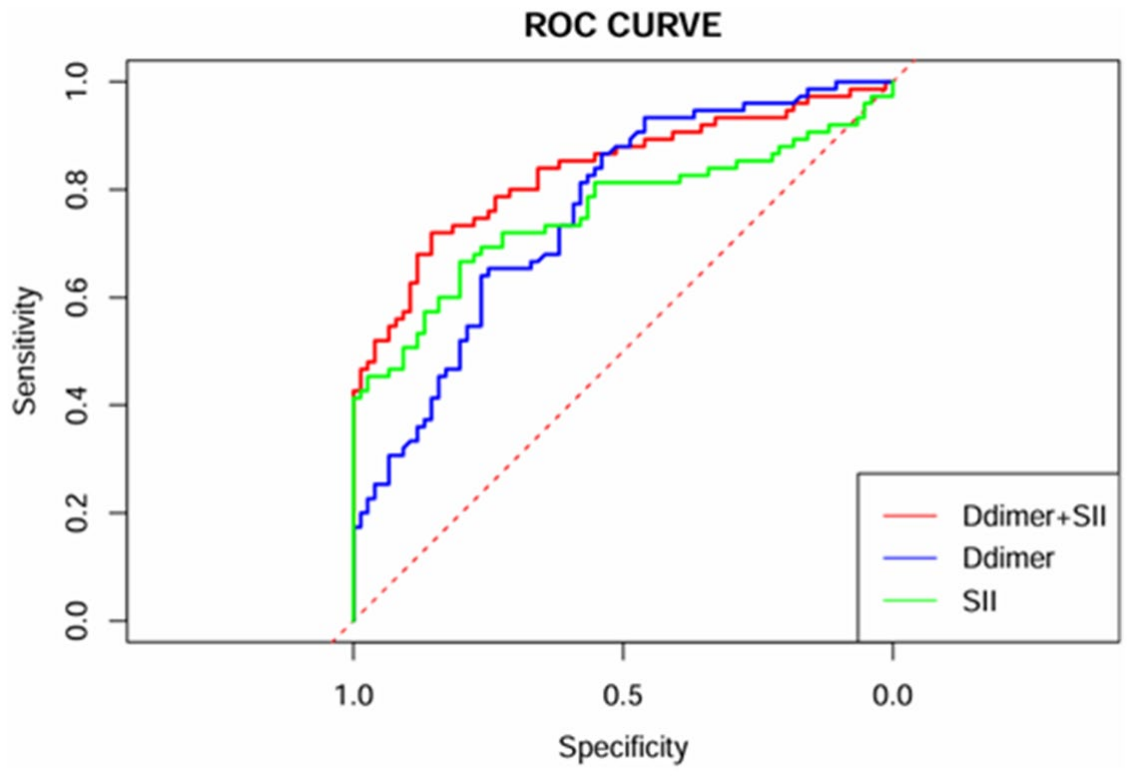
ROC curves of D-dimer, SII, and their combination.

ROC curve analysis was performed for the variables identified as independent risk factors for AECOPD with PTE in the multivariate logistic regression analysis. The results showed that for D-dimer, the AUC was 0.758 (95% CI: 0.682–0.834), with a cutoff value of 1.16 μg/mL, yielding a sensitivity of 0.867 and a specificity of 0.539. For SII, the AUC was 0.757 (95% CI: 0.677–0.838), with a cutoff value of 1496.66 × 10^9/L, producing a sensitivity of 0.667 and a specificity of 0.803. When combining D-dimer and SII, the predictive performance improved, with the highest AUC of 0.834 (95% CI: 0.768–0.900), a sensitivity of 0.720, and a specificity of 0.855. For further details, refer to Table 4 and Figure 1.

**Table 4:** Prediction value of D-dimer and SII for AECOPD complicated with PTE.

Variable	AUC (95%CI)	Cutoff value	Sensitivity	Specificity	*p* value
D-dimer (μg/mL)	0.758 (0.682–0.834)	1.16	0.867	0.539	<0.001*
SII	0.757 (0.677–0.838)	1496.66	0.667	0.803	<0.001*
D-dimer+SII	0.834 (0.768–0.900)	0.481	0.720	0.855	<0.001*

*: *p* < 0.05; AECOPD, acute exacerbation of chronic obstructive pulmonary disease; PTE, pulmonary thromboembolism; SII, systemic immune-inflammatory index.

### Bootstrap internal validation

**Figure 2:**
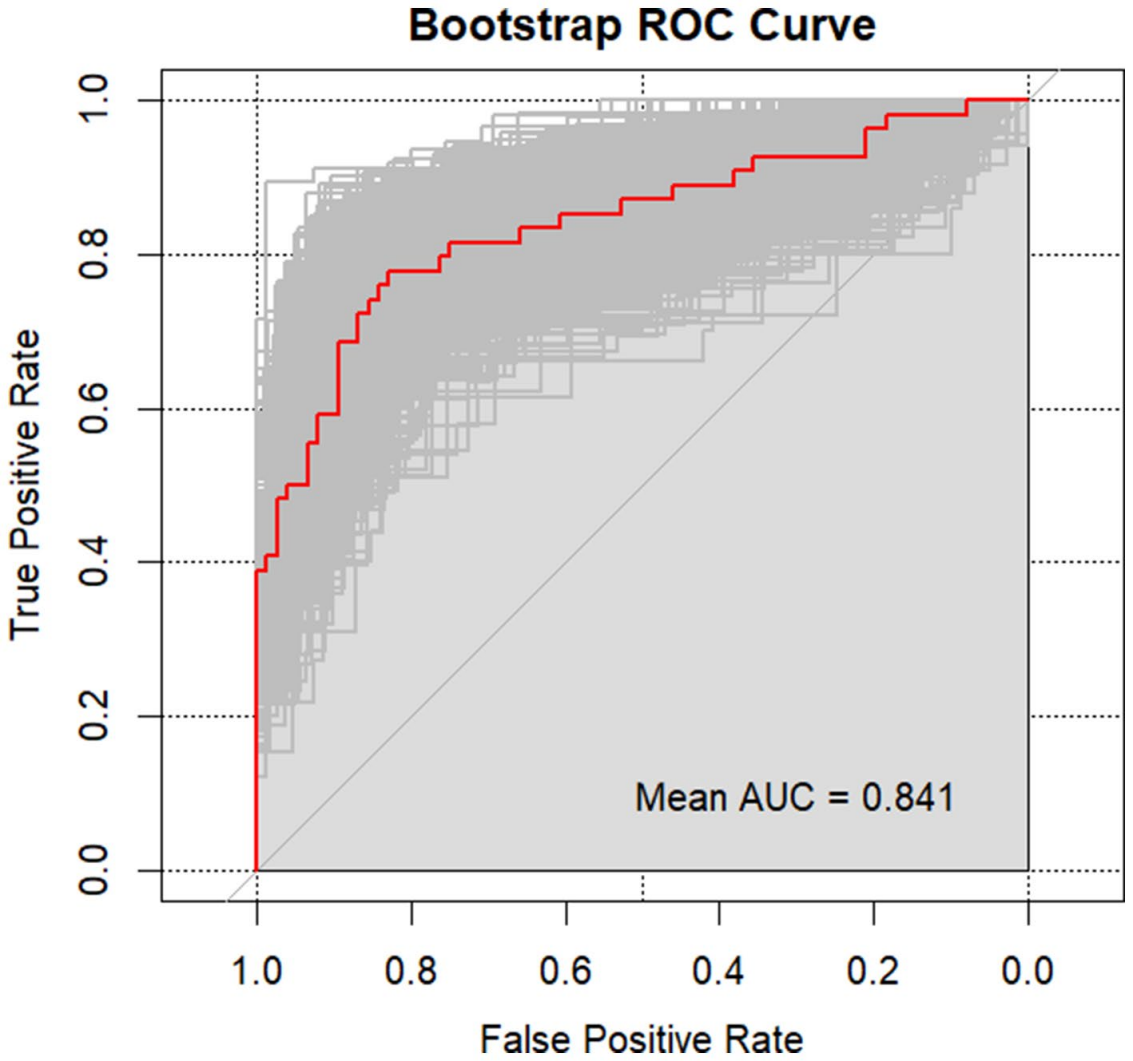
Bootstrap ROC curve of the combined model.

The results from the Bootstrap procedure revealed that the original AUC of the model which combined D-dimer and SII was 0.8409 (95% CI: 0.7649, 0.9156), with a bias of 0.0049 and a standard error of 0.0378. We have included the bootstrap ROC curve, shown in Figure 2, to further demonstrate the robustness and stability of the model. Additionally, a histogram of the bootstrap AUC distribution is presented as Supplementary Figure 1, illustrating the variability in model performance across the resampled datasets. The results demonstrated the robustness of the model.

### Spearman correlation analysis

Spearman correlation analysis was conducted to assess the relationships between D-dimer, SII, and NT-proBNP, high-sensitivity troponin T, and length of hospital stay. The results revealed that in AECOPD patients with PTE, both D-dimer and SII were positively correlated with the length of hospital stay, with correlation coefficients of *r* = 0.289 (*p* = 0.047) and *r* = 0.235 (*p* = 0.043), respectively. These correlations were statistically significant, though all demonstrated weak associations. Further details can be found in Table 5.

**Table 5:** Correlation analysis of D-dimer and SII in AECOPD patients complicated with PTE.

Variable	D-dimer		SII	
	*r*	*p*	*r*	*p*
D-dimer (μg/mL)	1.0	NA	0.008	0.948
SII	0.008	0.624	1.0	NA
NT-ProBNP (pg/mL)	0.025	0.259	0.078	0.506
High-sensitivity troponin T (ng/mL)	0.009	0.274	0.152	0.193
Hospital stays (days)	0.289	0.047*	0.235	0.043*

*: *p* < 0.05; AECOPD: acute exacerbation of chronic obstructive pulmonary disease; PTE, pulmonary thromboembolism; SII, systemic immune-inflammatory index; NT-proBNP, N-terminal pro-brain natriuretic peptide; NA, not applicable.

### Decision curve analysis

**Figure 3:**
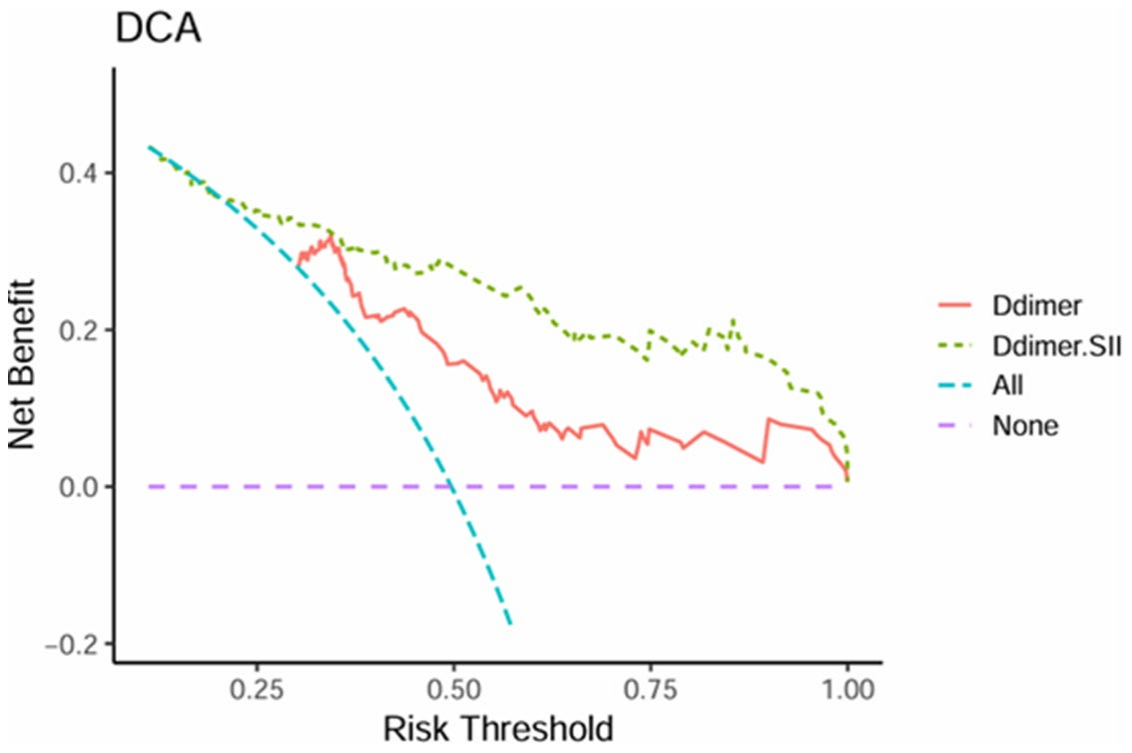
Decision curve analysis of D-dimer and D-dimer combined SII.

The net benefit of the D-dimer combined with SII model surpasses that of the D-dimer model across most risk threshold ranges in Figure 3, particularly at lower thresholds (0.25–0.5), indicating superior clinical utility. This suggests that at lower risk levels, the D-dimer combined with SII predictive model offers greater net benefit for clinical decision-making. At higher thresholds (0.75–1.0), the performance of the D-dimer model gradually converges with that of the D-dimer combined with SII model, demonstrating similar effectiveness at elevated risk levels. Overall, the D-dimer combined with SII model provides greater decision value across a broader range of thresholds, with significant advantages in low to moderate risk categories.

## Discussion

Our study revealed that patients with AECOPD and concurrent PTE exhibited significantly elevated levels of D-dimer and SII compared to those without PTE. The combined application of D-dimer and SII demonstrated superior predictive accuracy in identifying PTE among AECOPD patients. Significant differences were observed between PTE and non-PTE groups in inflammation status (such as white blood cell count, SII and SIRI), D-dimer, fibrinogen, urea, NT-proBNP, high-sensitivity troponin T, low-density lipoprotein and lower extremity venous thrombosis.

The clinical presentation of AECOPD can often mask concurrent PTE, rendering its detection particularly challenging. Our result illustrated that D-dimer, SII, lymphocyte count and lower extremity venous thrombosis were as independent predictors of PTE in this population. D-dimer, a fibrin degradation product, serves as a specific biomarker of fibrinolysis (19). Akpinar et al. proposed a revised D-dimer cutoff value to exclude the diagnosis of PTE in worsening COPD patients, establishing a cutoff of 0.95 μg/mL with an AUC of 0.752 (95% CI: 0.672–0.831) for diagnosing PTE (20). Consistent with the previous research results, our study also confirmed that D-dimer is an independent risk factor for AECOPD with PTE (21–23). However, when the cut-off value of the D-dimer in our study was 1.16 μg/mL and the sensitivity reached 0.867, it might still lead to a significant missed diagnosis of PTE in AECOPD. SII has emerged as a promising predictive marker for venous thromboembolism and is increasingly recognized as a novel inflammatory and prognostic biomarker for acute PTE (24, 25). A study by Gok et al. demonstrated that SII, which can be easily calculated from a complete blood count, is an independent predictor of extensive acute PTE and outperforms other inflammation-based markers (24). Our study found that SII was significantly higher in the AECOPD with PTE group compared to the AECOPD-only group, effectively predicting PTE presence among AECOPD patients. Additionally, we also demonstrate a weak correlation between SII and a longer hospital stay, suggesting that elevated SII levels may reflect a more severe condition in AECOPD patients with PTE, requiring prolonged hospitalization.

To further illustrate the predictive value of these markers, ROC analysis was conducted on D-dimer in conjunction with SII. The results unveiled an AUC of 0.834 (95% CI 0.768–0.900) for the combined use of D-dimer and SII in AECOPD patients with PTE Additionally, the sensitivity and specificity of the AUC were determined to be 72 and 85.5%, respectively, indicating the model’ s high accuracy in forecasting the occurrence of PTE among AECOPD patients. Furthermore, DCA confirmed that the combined use of D-dimer and the SII yields substantial net clinical benefit for predicting PTE in patients with AECOPD. The amalgamation of SII and D-dimer enables a more comprehensive assessment of PTE in AECOPD patients. SII signifies the systemic immune inflammatory response, whereas D-dimer reflect active coagulation and fibrinolysis. The amalgamation of these two aspects leads to a more accurate prediction of PTE. When imaging examinations such as pulmonary artery CTA cannot be performed, clinicians can quickly and simply assess whether patients with AECOPD have PTE through blood tests, which has important clinical significance and is expected to be applied in the future.

Our study has several limitations. First, it was a retrospective single-center study with a relatively small sample size, which may introduce selection bias and other inherent biases. Future prospective studies involving larger populations are necessary to validate our findings. Finally, many COPD patients receive long-term oral or inhaled corticosteroids to manage symptoms, and this study did not account for the potential impact of these steroids on blood biomarkers. The studies found that the use of oral corticosteroids and inhaled corticosteroids was associated with a reduction in inflammatory cells, leading to a decrease or stabilization of the SII, and this study did not account for the potential impact of these steroids on blood biomarkers (26–28).

In summary, both D-dimer and SII are independent risk factors for AECOPD with PTE, and their combination offers superior predictive value compared to either marker alone. Furthermore, elevated levels of D-dimer and SII were weak positively correlated with longer hospital stays, potentially indicating greater disease severity in AECOPD patients with PTE. This combined approach shows potential for future clinical applications. Based on these; to facilitate early identification of potential PTE in patients with AECOPD, clinicians should integrate D-dimer levels with the SII. Close monitoring of these biomarkers can provide critical insights for accurately predicting the occurrence of AECOPD complicated by PTE. We should validate the generalizability of the combined SII and D-dimer model through multicenter cohorts in future and conduct longitudinal studies to elucidate the mechanisms of “immunothrombosis” in acute exacerbation of chronic obstructive pulmonary disease with pulmonary thromboembolism (AECOPD-PTE). Randomized controlled trials are needed to evaluate precision anticoagulation strategies based on the combined SII and D-dimer model and to assess their health economic benefits in AECOPD-PTE. Furthermore, integrating multi-omics technologies to identify novel molecular targets will provide new insights into the early diagnosis and targeted treatment of AECOPD-PTE.

## Data Availability

The raw data supporting the conclusions of this article will be made available by the authors, without undue reservation.
