# Supplementary figures and images for: The predictive value of D-dimer combined with systemic immune-inflammation index for the presence of pulmonary thromboembolism in AECOPD patients
Source: Front Med (Lausanne). 2025 Aug 1;12:1582913. doi: 10.3389/fmed.2025.1582913 (PMC12354464; doi:10.3389/fmed.2025.1582913)

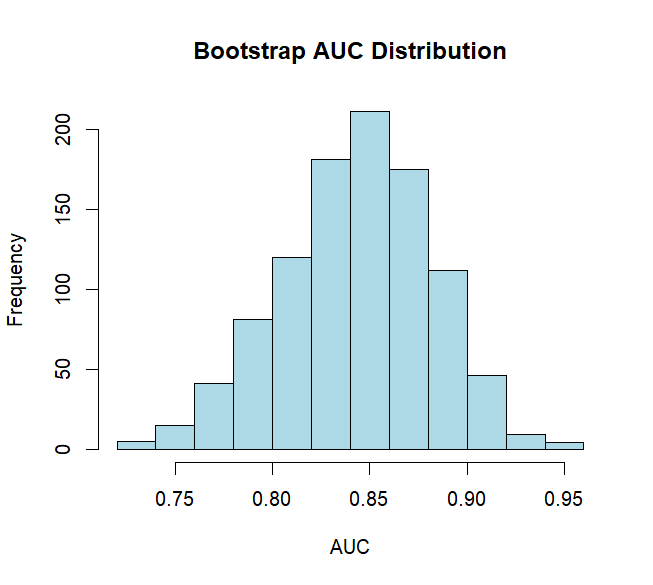

Supplement: Supplementary file 1 [file Image_1.tiff]
